# Mr Lambe's Report on Cancer

**Published:** 1810-10

**Authors:** Willm. Lambe


### *To the Editors of the Medical and Physical Journal.*

GENTLEMEN,

YOUR candour will, I doubt not, permit me to correct a great error into which Mr Royston has fallen, I dare say from inadvertence, in his account of my "Reports on the Effects of Regimen in Cancer," published in your Journal for July last.

After

After relating my recommendation of distilled water. Mr. Royston adds, " but he advises, as an auxiliary to this process, a diet of vegetables *in their raw state.*" This is advice with which it would be impossible to comply, in our climate at least. I therefore cannot suffer such a representation to pass unnoticed, as it tends to make the proposal ridiculous, and thereby to aggravate the prejudices with which it has to contend. My advice is really distilled water, a common vegetable diet (meaning thereby every thing eatable except flesh, fish, eggs, and much milk, and dressed in the common way); and I moreover think it useful, that *some fresh vegetable matter* should be taken daily. I need not say how different this is from Mr Royston's account of the regimen I recommended.

It is not my intention to enter into a defence of this proposal. It is before the public, and in due time the facts will speak for themselves. But I will simply say in its behalf, that the only gentleman who (as far as I know,) has tried the practice, has confirmed the facts on which I have founded its claim to a fair investigation. Two letters on the subject have appeared under the signature of a Dispensery Surgeon, in a contemporary journal, the Monthly Compendium of Medicine, &c. in which the writer, whilst he expresses a liberal dissent from all the conclusions I have drawn, has given the following direct testimony to its utility in scirrhous tumours. " In scirrhous tumours, where the patient's stamina is good, and particularly where the [illegible]terine secretion is regular, the vegetable diet and distilled water have proved very beneficial." He adds, very truly, " the good effects of Dr Lambe's treatment depend entirely on the natural stamina of the patient." In a second communication he has given the following testimony with regard to its utility in ulcers. " A vegetable diet and distilled water, by quieting the system, I have found very beneficial in checking the progress of cancerous and scrophulous ulcers." The observations he has made with regard to other diseases are so consonant with my own experience, that their accuracy cannot be doubted.

Observations such as these have laid the foundation of the system I have ventured to deliver. The very first case of cancer I treated (a very deplorable one) I had the opportunity of shewing to Mr. Abernethy, which I did that no doubt might exist as to the correctness of the observation. In the second, (a very large ulcerated cancer) which this gentleman was kind enough to put into my hands, the first impression of the distilled water upon the local disease, was so strongly marked, that Mr Abernethy, with his characteristic candour, declared, " I cannot be insensible to the effect of this treatment, I do not not doubt that it would prevent the disease; whether it will cure it or not I cannot say."

Persons with the best intentions and sufficient clearness of intellect, reason so differently from the very same data, that uniformity of sentiment cannot be expected, till a respectable body of evidence has been obtained. But let not palpable observations be disputed, unless overthrown by or rendered doubtful by contradictory observations. We can all have but one common object. Unhappily, however zealous we may be to promote science, or disseminate truth, the prejudices, ignorance, and perverseness of mankind form an obstacle to the regular conduct of the necessary experiments, and will probably for many years frustrate all attempts to ameliorate their condition. I am, Gentlemen,

Your obedient servant,

*September* 10, 1810. WILLm. LAMBE.

